# Analysing a private city being built from scratch through a social and environmental justice framework: A research agenda

**DOI:** 10.1177/00420980231211814

**Published:** 2023-12-15

**Authors:** Sarah Moser, Nufar Avni

**Affiliations:** McGill University, Canada; The Hebrew University of Jerusalem, Israel

**Keywords:** environmental justice, Forest City, Malaysia, new cities, privatised urbanisation, social justice, 环境正义, 森林城市, 马来西亚, 新城市, 私有化城市化, 社会正义

## Abstract

A growing body of scholarship examines new cities being built from scratch that are developed and governed by the private sector. While this scholarship explores discourse and rhetoric, economic objectives, and some social and environmental impacts of new private cities, scholars to date have not taken a social or environmental justice approach to analysing new city projects. In this article we examine Forest City, a private city project being built on artificial islands off the coast of Malaysia by one of China’s largest property development companies, and its unique governance and claims to being ‘eco’, despite the significant environmental damage it has caused. Intended as a lush and exclusive gated enclave for Chinese nationals, Forest City is a productive case study through which to consider the consequences of a private city using the frameworks of social and environmental justice. We suggest more critical research that engages with social and environmental justice is needed on the many emerging projects branded as eco-cities of the future, a troubling claim that signals a growing normalisation of mega-scale privatisation and loose or absent regulations regarding social inclusivity and environmental protection.

## 摘要

越来越多的学术研究探讨由私营部门开发和治理的、从零开始建设的新城市。虽然这方面的学术研究探讨了新的私有城市的话语和修辞、经济目标以及一些社会和环境影响，但迄今为止，学者们还没有从社会或环境正义的进路来分析新城市项目。在本文中，我们研究“森林城市 **(Forest City)**”项目及其独特的治理和“生态”主张，尽管它已经对环境造成了严重破坏。这个项目是由中国最大的一家房地产开发公司在马来西亚海岸的一个人工岛上建设的私有城市项目。森林城市旨在为中国公民打造一个郁郁葱葱的专属门禁飞地。它是一个富有成效的案例研究，通过它，我们可以使用社会和环境正义框架来研究私有城市的后果。我们认为，我们需要对许多被标榜为“未来生态城市”的新项目进行更多社会和环境正义方面的批判性研究。这种令人不安的标榜标志着大规模私有化的日益常态化，以及社会包容性和环境保护方面监管的松散或缺乏。

## Introduction

Over the past decade, there has been a global proliferation of new cities and urban mega-developments built from scratch that have heavy private sector involvement, and are intended as privately built and privately governed entities, politically and geographically separate from existing settlements. Such urban mega-developments can be characterised as entrepreneurial (Harvey, 1989), highly speculative (Goldman, 2011) and ‘fast’ (Datta and Shaban, 2017), and they are proliferating in Asia (Keeton, 2011; Moser, 2020), the Middle East (Cugurullo, 2018; Jeffrey, 2021; Moser et al., 2015) and Africa (Côté-Roy and Moser, 2019; Keeton and Provoost, 2019), with several failed or fledgeling projects in North America (Haggart and Spicer, 2022; Rebentisch et al., 2020). Private cities have features in common with gated communities, particularly in who can buy and access the space, the homogeneity of residents, social exclusions produced and how city builders tailor their projects for a wealthy clientele. However, there are some important differences in their scale and economic ambition, as builders of private new cities are entrepreneurs who aim to attract businesses and spark economic growth rather than primarily provide prestigious and securitised housing (Moser and Côté-Roy, 2021).

In this article, we focus on Forest City, a private, gated new city project in Malaysia to highlight a gap in the literature on new city projects, to think through issues of social and environmental justice, and to provide a framework for future analyses of private new city projects. Forest City is being built on four artificial islands by one of China’s largest property developers, with a target population of 700,000. Strategically located along one of the world’s busiest shipping channels and just two kilometres off the coast of Singapore, Forest City is the largest and most expensive Chinese real estate development outside of China. Announced in 2013, land reclamation activities started in 2014, and today much of the first island is complete, along with 65 condominium towers, over 200 luxury villas, a hotel, a private international school and a commercial area. Builders of Forest City have secured unique concessions of sovereignty from Malaysia, including the exclusive use of private security rather than Malaysian police and duty free status for the entire project, and the initial concept of the project was an elite racialised enclave for Chinese nationals (Moser, 2018) that was not obligated to abide by Malaysian racial quota requirements (Foo and Wong, 2014).

A small but growing body of scholarship examines private new cities, focusing particularly on their governance (Fält, 2019; Korah, 2020; Murray, 2015), eco claims (Cugurullo, 2016; Datta, 2012), economic ambitions (Moser et al., 2015) and how the rhetoric of climate change adaptation both masks and intensifies the marginalisation of poor residents living nearby (Ajibade, 2017). Only a handful of papers have been published on Forest City to date and focus on the ecological damage and the politics of urban greening in a speculative project (Koh et al., 2022; Moser and Avery, 2021; Rahman, 2017a), the geopolitical dimensions of a project controlled by Chinese actors along a strategic global trade route and next to Southeast Asia’s strongest economy (Moser, 2018; Strangio, 2020), and the ways in which the project has been forced to compromise and pivot in the face of lagging sales and local resistance (Avery and Moser, 2023a). While Forest City has stalled due to low investor interest and the COVID-19 pandemic, meaning its future is uncertain (Sani, 2022), given the speed at which the project was launched and the unique dynamic of it being a private gated project built by and for Chinese nationals in coastal Malaysia, and given the proliferation of similar private luxury real estate projects being built on artificial land in Asia, the Gulf and sub-Saharan Africa, there are significant issues relating to social and environmental justice that require unpacking.

In this article we argue that attention urgently needs to be paid to the myriad injustices that result from gated, private city-scale developments and recent scholarship on social and environmental justice can serve as a useful framework for analysing them. Forest City is a particularly productive case study through which to investigate social and environmental injustices that have facilitated the creation of the project, and those caused or exacerbated by the project – both inside and beyond the projects themselves – including an intensification of social exclusions based on ethnicity, religion and socio-economic class; the foreignisation of space and resources; and the impacts on Indigenous livelihoods and nearby ecosystems.

Our research draws on official promotional material, media coverage and criticism about the project, and visits to Forest City in 2017, 2018, 2019, 2022 and 2023. Site visits involved taking official tours of the Sales Gallery, watching on-site promotional videos, touring show flats, walking around the project and interviewing potential investors, sales staff, management, and residents of Forest City, as well as residents of nearby villages.

Following this introduction, we provide an overview of critical urban studies and planning scholarship on social and environmental justice and on the global proliferation of new city projects. Second, we provide an overview of Forest City as an aspiring elite enclave designed by and for Chinese nationals in Malaysia. Third, we examine Forest City’s absence of social and environmental justice in how the project was conceived by elites behind closed doors, its controversial location atop the country’s largest seagrass field, its private governance, the social exclusions created or exacerbated, and the impacts on Indigenous and Malay livelihoods and local ecosystems. We end with concluding thoughts about Forest City as emblematic of an emerging type and scale of private urbanisation that utilises mined sand to create artificial land, and call for further research that adopts a social and environmental justice framework to analyse other such urban mega-developments.

## Social and environmental justice frameworks and new cities scholarship

Over the past several decades, critical urban theory scholars have emphasised social justice as a significant normative concept and a criterion through which to evaluate urban developments and processes (Fainstein, 2010; Harvey, 1973; Lake, 2016). Environmental justice has similarly been used to study the unequal distribution of hazards and polluters in urban space and demand more equitable management of green amenities (Agyeman et al., 2016; Bullard, 2000). A major strand of the social and environmental justice scholarship has stressed the injustices that result from the planning profession’s operation under conditions of capitalist exploitation and deeply entrenched neoliberalism. However, this research overwhelmingly focuses on established cities. We suggest that a dual social and environmental justice lens is critical to illuminate the diverse manifestations of dispossession stemming from growth coalitions’ involvement in the planning and construction of new cities, exemplified in this paper through Forest City. These coalitions tend to exhibit a complete disregard for the social and ecological injustices that their projects induce and sustain.

### Social and environmental justice approaches to understanding urban development

The centrality of social justice to critical geography and urban planning scholarship and practice has been established over the past several decades. Social justice has been addressed from a variety of angles, including distributive, procedural, indigenous, spatial and legal, as well as integrative approaches that bridge different justice components (Avni and Fischler, 2020; Connolly and Steil, 2009; Dadashpoor and Alvandipour, 2020). The right to the city (Lefebvre, 1996), advocacy planning (Davidoff, 1965) and equity planning (Krumholz and Forester, 1990) are only a few examples of influential movements that have placed justice at the forefront and called for equitable distribution of resources and greater representation of marginalised groups in planning processes. These approaches have emerged in response to deep inequalities generated by neoliberal capitalism and technocratic planning that prioritise the built over the human environment (Brenner et al., 2011; Harvey, 1973). A social justice lens is essential to critically examine who benefits and who loses from urban development, whose voices are being heard or are absent from the planning process, and whether planning advances the interests of the ‘haves’ or the ‘haves-not’ and why (Arnstein, 1969).

Environmental justice also centres around notions of participation, access, and equity regarding environmental issues (Patsias, 2023) and has been similarly explored from distributional, procedural, legal and other lenses (O’Neill, 2023). Although intimately related, the two concepts – social and environmental justice – are often discussed separately in the literature (Avni and Fischler, 2020; Wessells, 2014). Environmental justice was initially developed in geography, planning, and sustainability scholarship to accentuate the disproportional proximity of racialised minorities and poor communities and their exposure to environmental hazards and polluters, such as toxic waste (Bullard, 2000). At the same time, these communities’ access to natural resources and green amenities such as parks and waterways, which are considered vital to health and wellbeing, has been limited and unevenly distributed in space (Rigolon, 2016). The environmental justice frame has been beneficial not only in identifying these disparities but also in mobilising communities around them and demanding change (Agyeman et al., 2016; O’Neill, 2023). Over time, the justice agenda has been broadened to include issues such as food security, energy and climate change (Anguelovski, 2013).

Social and environmental justice goals have been challenged in the neoliberal, capitalist system within which planning has been operating in the last few decades. Already in the mid-1970s, Molotch’s (1976: 309) ‘growth machine’ thesis argued that cities are driven by the interests of land-based elites that seek to profit from the intensification of an area’s land use; thus, ‘the political and economic essence of virtually any given locality … is growth’. The growth machine is propelled by a coalition of powerful interest groups, including real estate developers, business elites, entrepreneurs and political leaders who work in tandem to promote and sustain urban development for their own economic interests. This includes harnessing the national government to create favourable conditions for economic success (Logan and Molotch, 1976). This framework is useful for analysing new cities, as we later demonstrate.

Adopting an even more radical stance that considers also the ‘users’ of space, Harvey (1973, 2008) has consistently argued that the right to the city is unattainable under the capitalist system, where urban development is meant to serve private property owners by way of dispossessing the poor and marginalised. One example of a recent arena where justice principles might be jeopardised to incentivise development is smart cities (Datta, 2021; Yigitcanlar, 2021), which, similar to new cities (Moser and Côté-Roy, 2021), are officially proclaimed as a panacea to a host of social problems brought forward by rapid urbanisation. However, as critical scholars have shown, the smart city is a speculative economic project and a vehicle for property-led development, ‘the latest attempt to use and reconfigure the city as an accumulation strategy’ (Kitchin et al., 2019: 5). The smart city, thus, reproduces and even aggravates social and environmental injustices (Kitchin et al., 2019; MacKinnon et al., 2022; Sengupta and Sengupta, 2022). The smart city discourse is particularly relevant for private new city projects; however, the tensions between property-led-development and justice goals are ubiquitous.

‘Ecological’ initiatives such as greening and climate-mitigation projects have also been seen as part of the ‘growth machine’, appropriating and even compromising natural resources to gain financial interests and serve elite groups. A voluminous literature on green or environmental gentrification has highlighted how planners use green amenities and infrastructure such as waterfronts, bike paths and parks to tailor the urban space to the preferences of more desirable groups, at the expense of more marginalised ones (Anguelovski et al., 2019; Moser and Avery, 2021; Rigolon and Nemeth, 2018; Wolch et al., 2014). The very concept of environmental justice can be coopted by policymakers to justify projects with controversial environmental outcomes, such as desalination, claiming to serve the public good while being promoted to benefit the private sector (O’Neill, 2023). While the prioritisation of growth considerations is often deliberative, even when planners intend to promote environmental justice and equity, their actions sometimes lead to unsustainable and unjust consequences (Avni, 2019; Mandelbaum, 2021).

The seeming paradox between the positive health and wellbeing attributes associated with green amenities on the one hand, and how they are used to drive and perpetuate inequalities on the other hand, is a subject of burgeoning body of research. This scholarship provides a productive framework for analysing emerging private new city projects. We now turn to discuss how social and environmental justice relates to new cities.

### Private new cities: Social exclusions, environmental impacts

Over the past 15 years, there has been a proliferation of new city projects announced in around 45 countries, primarily in the Global South and emerging economies. While most of these are state initiatives or complicated public–private partnerships, some are entirely or primarily driven and financed by private actors. The scale of these private projects has tremendous and lasting impacts on ecosystems and their governance, and will have serious implications for both residents and nearby communities living in their shadows.

New cities dominated by the private sector are being built from scratch in Malaysia, India, South Africa, Ghana, Nigeria, Palestine, Saudi Arabia, Kuwait and more. These projects are largely designed, financed, and governed by the private sector and are a ‘key format that allows for a high degree of experimentation’, particularly in terms of governance and urban planning (Moser and Côté-Roy, 2021: 5). Private sector-driven new city projects are justified as a way to overcome the perceived inefficiencies of states and municipal governments, particularly their lack of overall vision, resources, capabilities and innovative and flexible urban management (Fält, 2019). Private city-scale projects are launched purportedly to fulfil a need for new forms of experimental governance and inability of governments to conduct such experiments. The managed retreat from government presence can perhaps best be seen in charter cities, a particular iteration of a private city driven by libertarian ethos and emerging most prominently in Honduras (Brustein, 2021; Lynch, 2019).

As private for-profit ventures, these city-scale developments are inherently entrepreneurial and prioritise profit over environmental or social sustainability (Datta, 2012), even promoting their social exclusivity to potential buyers as an attractive feature (Moser, 2020). In the case of Lavasa, a private city project in India, the lack of transparency, minimal legal enforcement and weak regulation have meant that there is little accountability to ensure the project lives up to its environmental claims (Datta, 2012). Private cities have a corporatised management structure and are headed by a CEO rather than an elected mayor and city council, meaning that there is no possibility for democratic governance (Moser, 2020). Conventional lines between public and private are further blurred when private new city projects are built as corporations listed on stock exchanges, an arrangement that means that city managers are beholden primarily to shareholders over residents (Moser et al., 2015). The private governance structures of Waterfall City in South Africa exemplify how public authorities have been replaced with private management and control, in an urban environment that is promoted as ‘smart’ and high tech, as well as one that conforms to conservative Muslim cultural norms, thus excluding non-Muslims and more moderate Muslims (Murray, 2015). Socio-economic exclusions are apparent in Eko Atlantic, a city extension of Lagos, that prioritises elites and further marginalises the poor living in nearby flood-prone communities (Ajibade, 2017). As an elite enclave for wealthy Nigerians, Eko Atlantic claims to address urgent flooding that plagues Lagos through its ‘Great Wall of Lagos’ seawall flood management scheme (Adelekan, 2016). However, while the seawall and urban extension will benefit Eko Atlantic and secure the project from the effects of climate change, it will intensify the vulnerability of nearby poor coastal areas, a process characterised as ‘climate apartheid’ (Acey, 2019; Amakihe, 2017).

The body of scholarship on new private cities is still in its infancy and much more attention needs to be paid to the various ways in which these speculative and entrepreneurial projects exacerbate or create new social divisions, and what new injustices are caused by both their environmental impacts and unprecedented speed of gaining approvals and construction.

## An absence of social and environmental justice in Forest City, Malaysia

Forest City’s fantastical and improbable design and the outrageously undemocratic way in which it was implemented demonstrate a shocking lack of concern for laws and consequences for breaking them, and for the damage to land, water, and livelihoods of Malaysians. Forest City reflects the sharp increase over the past decade in Southeast Asia in the financialisation of real estate and the ‘foreignisation’ of urban space (Fauveaud, 2020) that is shaped in large part by the rise of China’s Belt and Road Initiative. In many ways it represents a new type and scale of private elite urban mega-development with unprecedented social and environmental impacts. As a collaboration between one of China’s top property developers and the Sultan of Johor, Malaysia, Forest City completely lacked public input at every stage, a scenario that is common in both public and private new city projects, which are emerging overwhelmingly in authoritarian or undemocratic contexts globally over the past two decades (Moser and Côté-Roy, 2021; Watson, 2014). If we hope to prevent more Forest City-type projects, scholars need to demonstrate the myriad social and environmental injustices that plague them and which serve to sustain and exacerbate socio-economic difference and further entrench asymmetries of power.

### Social injustices in the ‘model city of the future’

The initial conceptualisation of Forest City took place among elites behind closed doors, skirting, or perhaps more accurately, ignoring local laws, and proceeding at such a fast pace that nearby residents and environmental groups were unable to mount an opposition of any significance (Avery and Moser, 2023a). As in other contexts of urban mega-developments in other parts of the world, speed is a key strategy to expedite construction processes and bypass modes of regulation (Côté-Roy and Moser, 2023; Cugurullo, 2017; Datta, 2017; Datta and Shaban, 2017). Locals whose livelihoods were to be affected were not notified and only learned of the project when they saw dozens of dredgers dumping sand in their fishing grounds in the Johor Strait. Land reclamation started in 2014 without a legally required Detailed Environmental Impact Assessment (Ourbis and Shaw, 2017). The way in which Forest City was given the green light exemplifies the power of growth coalitions to bypass local laws and norms, change land use designations and so on, a process Roy (2009) describes as ‘informality from above’, a contrast to how informality is conventionally conceptualised, namely as the poor illegally squatting on land they do not own. While the powerful are able to bypass local laws and expropriate land illegally, Indigenous villagers are routinely treated as illegal squatters or obstacles to development and progress as coastal land in Johor has become more attractive to developers. Indigenous communities have fought back in overt and covert ways, attempting to live their daily lives in the shadow of eviction threats (Bayat, 2000).

**Figure 1.**
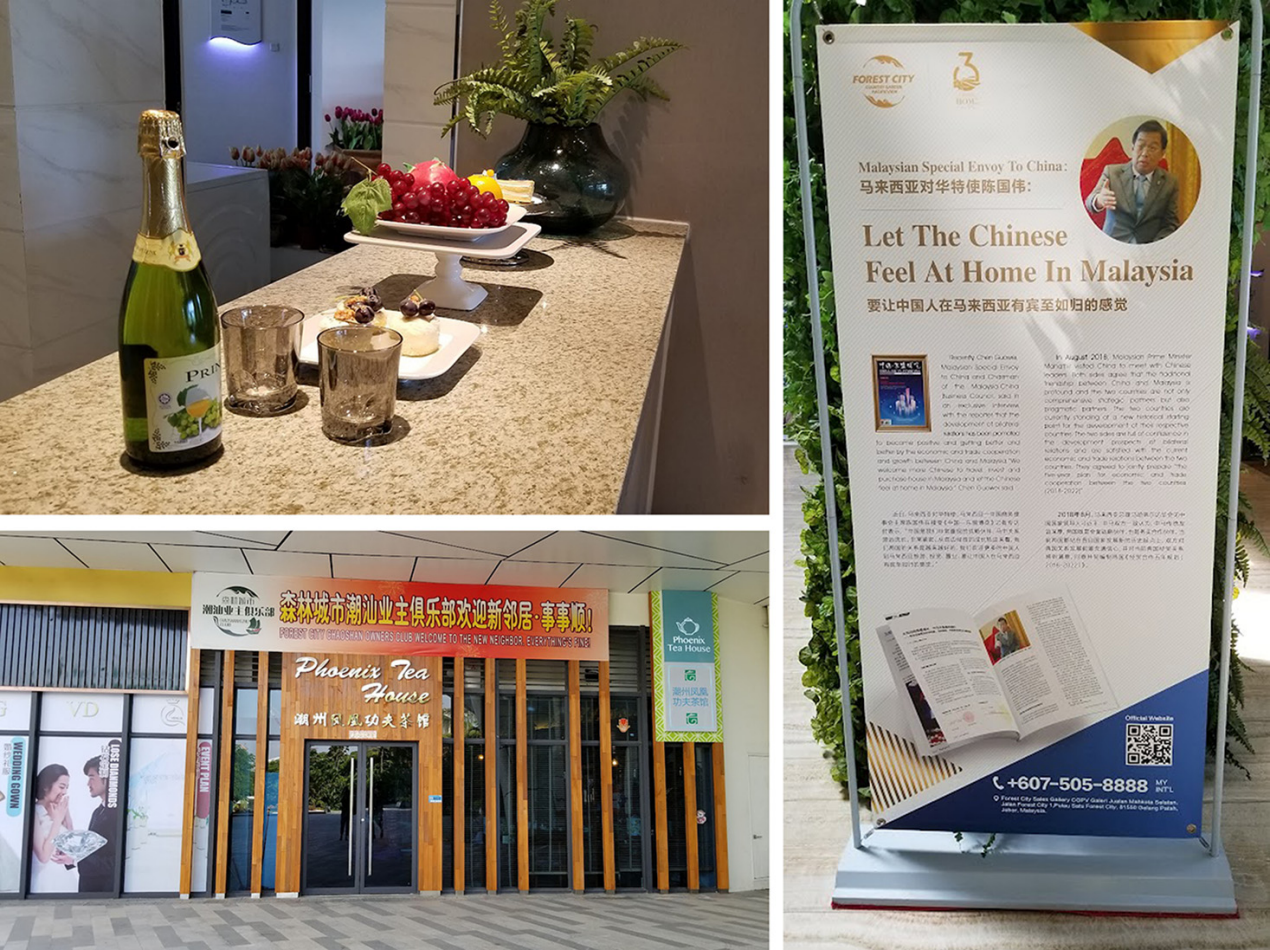
Throughout Forest City it is clear that ethnic Chinese buyers are the target. *Source*: Authors.

Forest City creates social exclusions based on ethnicity, religion, and socio-economic class and it is clear throughout the project that Chinese buyers are the target while Malay Muslims, who constitute the majority of nearby villagers and the majority of Johor’s population, are absent from promotional material. Signs throughout the project are written in Chinese, information about visa programmes feature prominently throughout the Sales Gallery, show flats are designed for Chinese tastes and feature mock alcohol, sales staff speak Mandarin, and the project’s slogans, such as ‘jade carved out of the ocean’, are written for a Chinese audience (Figure 1). Forest City’s only school, a branch of a Minnesota-based private school, offers only two languages: English and Mandarin. A year of tuition for a day student costs $24,000 – about four times the average annual wage in Malaysia (Mahtani, 2018). To date, only Chinese businesses have attempted to open in Forest City and there is a total lack of amenities and religious infrastructure for Muslims. Significantly, according to Google Maps, the nearest mosque is 23 minutes away by car or 1 hour and 11 minutes away on foot in a distant village, a significant inconvenience for those who choose to attend daily prayers. Due to Forest City’s intricate and high-maintenance decorative gardens, there is no room in the master plan for kitchen gardens, which are cherished by Malays as spaces to grow herbs and fruit trees (Moser and Avery, 2021). As such, Forest City marks the opposite of Malaysia’s official national goals of promoting social diversity, social inclusivity, and multiculturalism (Hamzah, 2021; Tan, 2020), which are also considered building blocks of the just city (Fainstein, 2010; Fincher and Iveson, 2012; Yiftachel et al., 2009).

**Figure 2.**
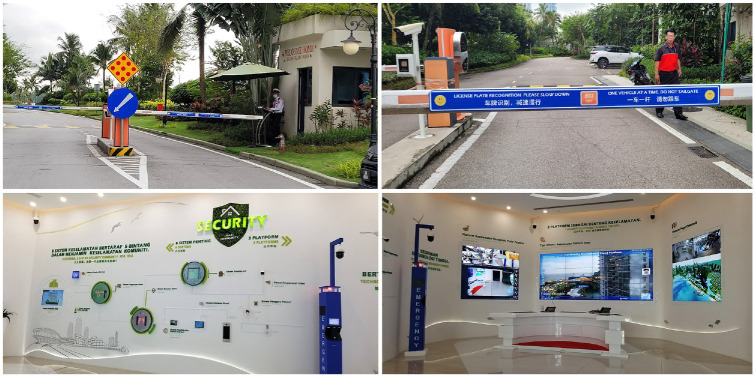
(a and b) Dozens of checkpoints throughout Forest City feature English and Mandarin, but significantly not Malay, the national language of Malaysia; (c and d) Smart security features in Forest City’s Visitor Gallery. *Source*: Authors.

Country Gardens, Forest City’s developer and China’s top property developer, negotiated various conditions that mark a contrast between Forest City and the rest of Johor, and even the rest of Iskandar Malaysia, the Special Economic Zone in which Forest City is located. The project is policed by private security guards from Nepal with questionable training and, just like security in a shopping mall, have the authority to expel people from the project for no reason. For example, one colleague who is an Indonesian Arab in her late 20s visited Forest City in 2017 with her cousin, who was heavily pregnant. After arriving, they sat down on the main beach near the Sales Gallery to rest. A security guard immediately blew a whistle at them and told them they were not allowed to sit on the beach or anywhere else. When they questioned this rule, they were told that they were being expelled from the project and they were not to return. According to our colleague, this was racial targeting as Chinese visitors were sitting on the beach and elsewhere, so they suspected they were not considered potential buyers and their presence was sending the wrong signal to Chinese visitors. ‘Public’ space in Forest City is not, in fact, open to the general public, and there is neither the practice nor the ideal of the commons in private Chinese developments. Locals are discouraged from utilising Forest City’s green spaces, including its waterfront area, which is typically considered public domain in virtually every waterfront redevelopment project around the globe (Moser and Avery, 2021). The different living compounds in Forest City are also guarded and secured, with checkpoints located throughout the project, and even we, as western foreigners, could not walk around freely (Figure 2a and b).

According to several sales staff, the number one fear of potential buyers from China is being ‘surrounded by’ Muslims, who they perceive as potentially violent and intolerant (tour of Sales Gallery taken in 2018). A second racialised fear of potential buyers from China is of Indians, who many perceive as ‘always drunk and misbehaving’ (tour of Sales Gallery taken in 2023). The developer has responded to the security concerns of potential investors with three ‘smart’ security platforms to ‘shield the city’, including electric fencing, a 3D security management cloud platform, and an ‘integrated security management platform’, which includes eight systems to ensure a ‘smart and safe city’ (Figure 2c and d). The use of AI technology, which itself has been criticised as replicating real-world racism (Photopoulos, 2021), and other digital safety features designed by Huawei, China’s largest ICT provider, are slated to securitise the project and detect threats, particularly Muslim threats against Chinese residents (Moser, 2018). Whether these features are built as planned remains to be seen, but the aspiration to include ‘smart’ technology to exclude undesirables and protect wealthy residents and the racialised fear embodied in Forest City is deeply concerning for access to the site. The gatedness of Forest City negatively impacts nearby residents, who do not have legal access to its amenities and might feel like second-class citizens as a result. Residing in Forest City is not simply a matter of housing affordability, which is in itself a justice issue (Avni, 2019; Muñoz, 2018), but is explicitly and implicitly dependent on one’s nationality, ethnicity or race, a classic example of a ‘fortress city’ (Low, 1997).

In a similar vein, while Forest City has won awards for its claims to be building affordable housing (Avery and Moser, 2023b), it has failed to produce any or even a substantive plan to open up the project to a broader swath of Malaysian society. Employees of Forest City have stated that ‘it is clear that the price of the property is too high for Malaysians, and [we have] specifically marketed towards the Chinese’ and admitted that ‘the people who are working in the area probably can’t afford to live here’ and ‘the majority of our buyers are foreign’ (Mahtani, 2018). While cities all around the world struggle with issues of diversity and affordability, Forest City makes no attempt to be inclusive and, in fact, broadcasts its exclusivity as a selling point. Residents of Johor confirm their exclusion, with one Johorian simply stating: ‘Forest City was not built for us’ (Interview, 2018). Finally, aspects of social justice concern not only the project’s targeted population but also the city builders. Forest City, much like many other new private cities built from scratch, has been constructed by foreign migrant workers from Bangladesh, India, Pakistan, Indonesia, and other Asian countries. Malaysian economic development in particular has become progressively reliant over the past several decades on the ‘constant availability of an invisible, highly diverse, fragmented, disposable pool of migrant non-citizen labour’ (Muniandy, 2020: 2294). Migrants working in the construction sector suffer dehumanising living conditions and tend to be housed in ramshackle camps without proper light, ventilation or privacy (Muniandy, 2021) while also facing dangerous working environments and even deaths. In the evenings, the movement of migrant workers is restricted by guard posts set up by Forest City management, and many workers were irregularly paid and even raided a nearby island for food (Rahman, 2017b). Due to their precarious status, they make do with this predicament while they construct the ‘smart’, ‘safe’ and ‘ecological’ city of the future. The juxtaposition of the labour camps with the luxurious condos and high-rises of the newly built city reveals yet another aspect of stark injustice.

### Environmental injustices of an eco-city

The environmental impacts of Forest City far exceed the physical boundaries of the project. The project is located atop Malaysia’s largest seagrass field, a rich and sensitive ecosystem that provides habitat for endangered species including seahorses and dugong (Hossain et al., 2019) and that had been earmarked for environmental protection (Avery and Moser, 2023a). The land reclamation activities, which started without the legally required Detailed Environmental Impact Assessment (Ourbis and Shaw, 2017), have had a serious impact on local ecosystems and Indigenous and Malay rural livelihoods, with sedimentation dramatically reducing yields from mussel farms, prawning activities in nearby mangroves, and fishing (Rahman, 2017a). The ongoing prioritisation of city-centric economic development neglects and marginalises the poor, who arguably have longer and more compelling claims to the land and have a far smaller ecological footprint, in favour of the wealthy foreign investors and the intended population.

**Figure 3.**
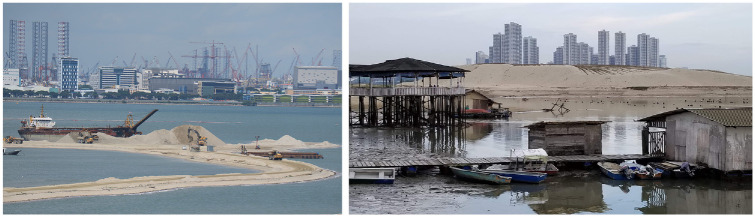
Dredging and land reclamation activity in and around Forest City. *Source*: Authors.

Forest City has the potential to significantly impact a variety of environmental aspects inland in the state of Johor. The project can be understood to have a parasitic relationship with the state of Johor, upon which it is wholly dependent for water, energy, food, garbage disposal, cemeteries/columbaria, labour and more. Forest City’s lavish decorative gardens, swimming pools and plans for luxury condos with pools on each floor completely overlook that Johor has been prone to droughts in recent years (Chuah et al., 2018). Sand for the project is being dredged from the sea and mined from islands and river beds around the Malay Peninsula, wreaking environmental damage far from the location of Forest City (Arnez, 2021; Beiser, 2017; Peduzzi, 2014; Figure 3). Beyond the lack of justice for nearby residents, the project also casts a long shadow on the long-term sustainability of Malaysia’s natural resources.

Finally, the exclusion of locals from the project, both as residents and visitors, described in the previous section, also has implications on environmental justice aspects due to the significance of access to green spaces for health and wellbeing (Avni and Fischler, 2020; Wolch et al., 2014). Projects such as Forest City allocate the many positive attributes of Malaysia’s coastal environment to a select few over the public at large, similar to green gentrification trends, but on a much larger scale. Nearby communities are not only burdened with the destruction of their local habitat but are also prevented from enjoying the natural amenities in their vicinity (Smardon et al., 2018) and green space in Forest City itself.

While Forest City claims to be a futuristic eco-city and a model for future urban development, it has smothered and destroyed most of the sea grass field upon which it is built and damaged surrounding mangroves (Rahman, 2017a). It does not attempt to recreate a mangrove ecosystem, but is filled with high-maintenance, manicured, purely decorative gardens with predominantly imported species from Africa, Latin America, and elsewhere (Moser and Avery, 2021). The city design is also oriented towards private car use, with wide lanes, elevated pedestrian passes, and no sidewalks on most streets, rendering it totally unwalkable. While claiming to be an ‘eco-city’, Forest City is failing to meet even basic sustainability standards.

## Justice in a ‘model city of the future’? Concluding thoughts

The emergence of a private, gated urban mega-development in the ocean such as Forest City is a troubling new form and scale of speculative urbanism (Goldman, 2011), the implications of which urgently require attention from scholars, policy makers, and the public. We use the Forest City case study as a call for social scientists to investigate the global proliferation of ex nihilo development through a social and environmental justice lens, particularly since most such projects are emerging in authoritarian contexts with little freedom of the press and where local scholars may fear retaliation for writing critically on high visibility projects (Moser and Côté-Roy, 2022).

This article seeks to contribute to social and environmental justice and new cities scholarship. While scholars have noted the deep inequalities generated by neoliberal capitalism and technocratic planning (Brenner et al., 2011; Harvey, 1973), they have considered established cities as their reference point. Yet new private cities add critical dimensions of injustices in their scale, modes of governance, and financial mechanisms, and, most importantly, they are key sites of contemporary urbanisation. Thus far, the limited scholarship on new private cities seems to suggest that they fail on every justice front: democracy, participation, inclusion, social and environmental sustainability, and diversity are simply absent from their planning and execution despite the sophisticated rhetoric and renderings used to promote these projects.

Forest City appears to be an extreme and uniquely audacious example of a private foreign city being built in the ocean, yet the construction of private urban mega-developments on artificial land is an accelerating trend in Asia, the Middle East and Africa due to the profitability and financialisation of real estate, the growing demand for securitised and elite spaces, and the increasing affordability and ease of land reclamation as opposed to land grabbing. These developments are the product of powerful growth coalitions that coalesce for the purpose of capital accumulation, with little or no regard for the adverse social and environmental outcomes. Some projects have recently stalled or collapsed due to China’s restrictions on outflows of capital, an oversupply of housing, and the COVID-19 pandemic. For example, Gateway Melaka, a new Chinese city project and seaport off the coast of Melaka on three artificial islands slated to be the largest artificial island project in Southeast Asia, was launched in 2014 and cancelled in 2021 (Kumar, 2020). The project devastated the fishing grounds of the Kristang, a 500-year-old Portuguese settlement in Melaka (Arnez, 2021; Beech, 2020; Low, 2016) while undermining the cultural and historical significance of Melaka as a coastal city designated as a UNESCO World Heritage site (Connolly, 2023).

Despite the environmental devastation and the collapse of these projects, new mega-developments on reclaimed land continue to be announced (Table 1), including City of Pearl, an elite Chinese mega-development on artificial islands planned for the harbour of Manila (Cheng, 2017); the Maharani Energy Gateway, an offshore energy storage facility, near Muar, Malaysia (SCMP Reporters, 2022); five new city projects on reclaimed land in Bahrain, expanding the country’s land mass by 60% (Ebrahim, 2021); and the Red Sea Project, a massive coastal resort mega-project underway in Saudi Arabia (Narayanan, 2022). Yet only limited scholarship to date has explored the multiple injustices these projects are expected to bring. A joint social and environmental justice perspective is useful to unpack the various forms of violence, dispossession, and inequality that new city projects create and perpetuate.

**Table 1.** Private mega-developments and new city projects on artificial land.

Name of project	Location	Date announced	Projected population	Size	Status
Durrat al Bahrain	Bahrain	2002	60,000	5 km^2^	Nearly complete
Colombo International Financial City	Colombo, Sri Lanka	2011	80,000	2.69 km^2^	Underway
Ocean Flower Island	Hainan, China	2012	200,000	3.81 km^2^	Underway
Forest City	Johor, Malaysia	2013	700,000	13.7 km^2^	Partially complete, stalled
Melaka Gateway	Melaka, Malaysia	2014	Unknown	5.5 km^2^	Cancelled/on hold
City of Pearl	Manila Bay, Philippines	2017	500,000	4.1 km^2^	Proposed
Red Sea Project	Saudi Arabia	2017	1 million visitors/year	28,000 km^2^	Underway
Maharani Energy Gateway	Muar, Malaysia	2022	Unknown	13.7 km^2^	Proposed

*Source*: Authors.

Further work needs to be done on the legal contexts of projects such as Forest City to understand how local laws inequitably distribute justice in support of private investors and state actors. International investment law, property law and the legacies of colonial British law all need to be disentangled to understand how legal regimes tip the balance in favour of projects such as Forest City while denying rights and access to justice for poor locals. Future research could also productively interrogate what ‘smart’ refers to in the context of new city projects, what technologies are being employed, and how these technologies are at odds with creating a democratic and equitable society.
